# High‐dose Vitamin B6 supplementation reduces anxiety and strengthens visual surround suppression

**DOI:** 10.1002/hup.2852

**Published:** 2022-07-19

**Authors:** David T. Field, Rebekah O. Cracknell, Jessica R. Eastwood, Peter Scarfe, Claire M. Williams, Ying Zheng, Teresa Tavassoli

**Affiliations:** ^1^ School of Psychology and Clinical Language Sciences University of Reading Reading UK

**Keywords:** anxiety, depression, GABA, surround suppression, Vitamin B12, Vitamin B6

## Abstract

**Objective:**

Vitamins B6 and B12 are involved in metabolic processes that decrease neural excitation and increase inhibition. This double‐blind study investigated the effects of supplementation for 1 month with a high‐dose of B6 or B12, compared to placebo, on a range of behavioural outcome measures connected to the balance between neural inhibition and excitation.

**Methods:**

478 young adults were recruited over five linked phases. Self‐reported anxiety (*N* = 265) and depression (*N* = 146) were assessed at baseline and after supplementation. Several sensory measures acted as assays of inhibitory function and were assessed post‐supplementation only; these were surround suppression of visual contrast detection (*N* = 307), binocular rivalry reversal rate (*N* = 172), and a battery of tactile sensitivity tests (*N* = 180).

**Results:**

Vitamin B6 supplementation reduced self‐reported anxiety and induced a trend towards reduced depression, as well as increased surround suppression of visual contrast detection, but did not reliably influence the other outcome measures. Vitamin B12 supplementation produced trends towards changes in anxiety and visual processing.

**Conclusions:**

Our results suggest that high‐dose Vitamin B6 supplementation increases inhibitory GABAergic neural influences, which is consistent with its known role in the synthesis of GABA.

## 1 INTRODUCTION

B vitamins play an integral role in a large number of anabolic and catabolic cellular processes that are essential for nervous system and brain function, including several that help to maintain an appropriate balance between neural inhibition and excitation by up‐regulating inhibition and down‐regulating excitation. This is an important role because an equilibrium shifted too far towards excitation has been implicated in a number of neuropsychiatric disorders, including anxiety and depression (Luscher & Fuchs, 2015; Sohal & Rubenstein, 2019). In this context it has been proposed that high‐dose B vitamin supplementation might be an effective strategy for enhancing behaviourally observable effects of neural inhibition, and for damping down excessive neural excitation (Holton, 2021). Consistent with this proposal, Kennedy (2016) argues that the optimum level of dietary B vitamins has not yet been established, but it certainly exceeds the Recommended Daily Allowance (RDA) and many individuals are borderline deficient.

The first study we are aware of that proposed increasing neural inhibition in typical adults through B vitamin supplementation was carried out by Smith et al. (2017). They supplemented participants with B vitamin rich Marmite or a control and measured steady state visual evoked potentials (SSVEPs) while participants viewed visual stimuli designed to activate both excitatory and inhibitory mechanisms in the visual cortex. They found that SSVEP amplitudes were reduced following supplementation, implying an increase in neural inhibition and/or a reduction in excitation in visual cortex. To explain this, Smith et al. pointed to the well‐established role of Vitamin B6 as a coenzyme in the synthesis of the inhibitory neurotransmitter GABA from glutamate (Holtz & Palm, 1964; Martin, 1987). However, other mechanisms of action cannot be ruled out because Marmite contains high levels of Vitamin B12 and other B vitamins that have their own potential mechanisms of action, as well as glutamate and tryptophan (a serotonin precursor), and furthermore the B6 levels in marmite are not especially high. While we believe Smith et al.‘s results may reflect an additive or synergistic effect of the active ingredients, here we report a direct test of the hypothesis that high‐dose Vitamin B6 supplementation can influence behavioural outcomes related to neural inhibition and the overall level of excitation. Such an influence can be predicted because in addition to its role as a coenzyme in converting excitatory glutamate into inhibitory GABA, Vitamin B6 is involved in a number of other pathways that are likely to reduce neural excitation; it is a co‐enzyme for the production of other neurotransmitters such as serotonin, dopamine, and noradrenaline; it acts as a cofactor in the kynurenine pathway in which it reduces the amount of quinolinic acid, which is an agonist to the excitatory NMDA receptor (Curto et al., 2015; Zinger et al., 2011) it is involved in the homocysteine‐cysteine cycle and through this reduces levels of homocysteine, which is an agonist of the NMDA receptor (Deep et al., 2019; Zaric et al., 2019) also via the homocysteine‐cysteine cycle it provides cysteine to the glutathione cycle, which reduces levels of the excitatory neurotransmitter glutamate by converting it to glutathione.

Vitamin B12 is able to substitute for B6 in the homocysteine‐cysteine cycle, and so shares two of the potential excitation reducing pathways described above with Vitamin B6. But additionally, Vitamin B12 may indirectly influence the balance via its role in maintaining the integrity of myelin (Shane, 2008). This possibility is suggested to us by the recent discovery that a large proportion of neocortical myelin ensheathes the axons of local inhibitory neurons (Micheva et al., 2016, 2018; Stedehouder et al., 2017). These findings contrast with the traditional view of myelin as mostly ensheathing excitatory long range axons, and contribute to a growing consensus that the role of myelin in the brain is more complex and dynamic than previously thought (Fields, 2008). Such local inhibitory neurons are responsible for the inhibitory mechanisms engaged in the SSVEP paradigm used by Smith et al. (2017), and by the visual paradigms used in patient groups discussed below. Therefore, if B12 preferentially influenced the myelination of this type of neuron, the overall strength of inhibition relative to excitation would be increased. Given this and the other two potential pathways identified, we also tested the possibility that high‐dose Vitamin B12 supplementation can influence behavioural outcomes related to neural inhibition and the overall level of excitation.

Atypicality of the inhibition‐excitation balance, and particularly of GABAergic activity, has been linked to anxiety (Nemeroff, 2003), depression (Kalueff & Nutt, 2007; Luscher & Fuchs, 2015), autism (Puts et al., 2017; Robertson et al., 2016), and schizophrenia (Lang et al., 2007). Visual and other sensory disturbances are experienced in many of these conditions, and these are thought to be related to excitation‐inhibition imbalances in the visual cortex. This has been established by measuring the effects of flanking surround stimuli on the ability of patient groups to perform visual tasks such as detecting a low contrast target or determining the direction in which a briefly presented target is moving (Yazdani et al., 2015). In neurotypical adults, depending on task specifics, flanking stimuli are found to diminish or enhance performance and these effects have been linked to GABAergic lateral inhibition in the visual cortex (Katzner et al., 2011; Moutsiana et al., 2011; Zenger‐Landolt & Heeger, 2003). This is often referred to as ‘contextual modulation’. In patient groups, effects of flanking stimuli are found to be either absent or modified compared to matched controls, and it is thought that this is caused by altered GABAergic function. Some of the more studied examples include depression (Golomb et al., 2009; Salmela et al., 2021), autism (Flevaris & Murray, 2015; Foss‐Feig et al., 2013), schizophrenia (Yoon et al., 2009, 2010), epilepsy (Schach et al., 2021; Yazdani et al., 2017), migraine (Battista et al., 2010, 2011), as well as ageing (Betts et al., 2005; Pitchaimuthu et al., 2017). These findings raise the interesting, currently largely unaddressed, question of whether dietary modulation of the excitation‐inhibition balance might modulate visual task performance, or prove to be therapeutic for patient groups. To begin addressing this question, we have included an appropriate visual task in our test battery to discover if the flanking/surround effect in neurotypical adults is modified by supplementation with either Vitamin B6 or B12.

Considering the involvement of the inhibition‐excitation balance in anxiety and depression, and the potential mechanisms of action of Vitamins B6 and B12 described above, suggests that Vitamin B6 status should be associated with mood related outcomes. A similar suggestion was made by McCarty (2000) who hypothesized that high dose B6 supplementation could be used as an ‘anti‐stress’ strategy. Consistent with these ideas, Mikkelsen et al. (2018) found that adults who habitually consume B vitamin rich yeast based spreads such as Marmite report lower anxiety and stress than those who do not, and Kafeshani et al. (2020) found that higher dietary B6 intake predicted reduced anxiety and depression risk in women but not men. Turning to studies that have supplemented with B6 or B12 these have generally recruited healthy older adults and have focused more on cognition than mood, and have produced mixed results. Dangour et al. (2015) conducted a B12 randomised controlled trial (RCT) in participants aged 75 and over with moderate B12 deficiency, and found no effect on peripheral and sensory nerve conduction, or cognitive function. On the other hand, Bryan et al. (2002) supplemented female participants spanning a wide age range with either B12, B6, or folate and found small positive effects of all three on memory, but not other aspects, of cognition or mood. Deijen et al. (1992) supplemented healthy males aged 70–79 with B6 for 3 months and found improved memory performance, but no effects on mood or other cognitive tasks. A second group of supplementation studies can be identified that have measured stress, anxiety and depression after supplementing adults with tablets whose major ingredient consists of several different B vitamins but which also contain other vitamins and minerals. Two meta‐analyses of these studies have both reached promising conclusions; Long and Benton (2013) found positive effects of multivitamins on stress, anxiety and mild psychiatric symptoms (but not depression) that were greater when the supplement contained a higher B vitamin content, while Young et al. (2019) selected for meta‐analysis only studies in which the supplement contained three or more B group vitamins and found a positive effect for stress but not anxiety or depression. Raising the question of whether Vitamin B6 was the key ingredient driving the positive results, Benton et al. (1995) found that the mood improvement effects were selectively associated with the B6 and Riboflavin status of participants. To address this question, the present study will test directly for effects of supplementing with a high dose of Vitamin B6 or B12 on self‐reported anxiety and depression symptoms.

Here we report the combined results of linked exploratory double‐blind placebo‐controlled trials carried out over several years that examined the individual effects of both Vitamin B12 and B6 supplementation compared to placebo on a general adult (mainly student) population. The doses we used were high relative to the RDA; for Vitamin B6 the RDA for adults aged 19–50 is 1.3 mg and the supplement contained 100 mg; for Vitamin B12 the RDA is 2.4 μg and the supplement contained 1000 μg. Unusually, we measured outcomes that span different domains: psychiatric symptoms, visual processing, and cognition. These were selected for their relevance to the hypothesis that Vitamins B6 and B12 may exert an effect via modification of the inhibition‐excitation balance. The outcome measures reported here are anxiety (SCAARED), depression (Mood and Feelings questionnaire (MFQ)), the ability to detect low contrast visual targets with and without a suppressive background pattern, the binocular rivalry reversal rate which has previously been linked to GABA levels in visual cortex, and a tactile sensory processing battery that has previously been used in relation to proposed inhibition/excitation imbalances in ASD. Recently, data collection was moved online due to the COVID‐19 pandemic and this necessitated modification of the test battery due to the technical limitations of online testing. Due to these modifications, and because online data collection is ongoing, a follow up manuscript will report additional results for a Go No Go task measuring cognitive response inhibition, measurements of the magnitude of visual illusions related to GABA levels (Tilt and Ebbinghaus illusions), Heart Rate Variability, the Depression Anxiety And Stress Scale, the Autism Quotient, Sensory Processing Inventory, ADHD questionnaire, Attentional Control Scale, and sleep quality (PSQI), as well as updating results for the SCAARED anxiety questionnaire which is also included in the ongoing online version of the study. The online phase of data collection also introduced three additional covariates which will be reported in the follow up manuscript, intended to model variation in performance due to daily variations in mood, sleep quality and alcohol consumption.

## 2 MATERIALS AND METHODS

### 2.1 Participants

The experiment was conducted over the course of 5 years by successive cohorts of BSc and MSc student experimenters. Participants were recruited in exchange for course credit, via adverts, word of mouth, and social media. In this way, a total of 478 participants (381 female, 92 male, 5 missing data; aged 18–58 years, mean 23.0, median 20, SD 7.2) were randomised to receive either Vitamin B6, Vitamin B12, or placebo tablets. However, due to technical problems that resulted in some missing data and also because no single outcome measure was included in all phases of data collection the number of participants completing each outcome measure was in all cases less than the full sample size. Details of the sample sizes for each outcome measure and the consequent effect sizes that would be detectable with an acceptable power of 0.8, assuming an alpha of 0.05 and two‐tailed tests, are given in Table 1. Some outcomes were measured at baseline and post‐test and some at post‐test only. For measures assessed at both time points, Table 1 also presents information on the number of participants completing both time points.

**TABLE 1:** Sample sizes for each outcome measure

Outcome measure	Baseline sample size (B6/B12/placebo)	Post‐test sample size (B6/B12/placebo)	Total sample size for main analysis after all exclusions	Effect size detectable with power = 0.8 (after all exclusions)
Anxiety: SCAARED	107/108/96	98/96/91	88/90/87	0.30
Depression: MFQ	52/52/54	52/51/53	47/48/51	0.41
Visual contrast detection	N/A	109/115/115	101/103/103	0.39
Binocular rivalry	N/A	56/59/62	56/58/58	0.52
Tactile reaction time	N/A	77/78/83	74/74/79	0.45
Tactile rtm. intrasubject variability	N/A	77/78/83	74/76/78	0.45
Tactile threshold	N/A	77/78/83	76/76/81	0.45
Tactile dynamic threshold	N/A	74/76/81	56/58/67	0.51
Dynamic threshold as % of static	N/A	75/76/82	56/58/66	0.51
Tactile single site adaptation threshold	N/A	75/77/81	72/76/77	0.46

*Note*: N/A in Baseline sample size indicates the measure was only made at post‐test. For measures that were collected at baseline and post‐test the effect size detectable is based on a paired samples t‐test comparing the two time points within the B6 group, two‐tailed, alpha = 0.05. For measures collected only at post‐test the effect size detectable is based on an independent samples t‐test comparing placebo to Vitamin B6.

Exclusion criteria were lactose intolerance (placebo tablets contained lactose), diabetes, epilepsy, and taking medications known to interfere with B vitamin absorption. Participants were asked if they were taking multivitamins that contained B vitamins, and were excluded unless they agreed to stop taking them for the duration of their participation in the study.

This study was conducted in the School of Psychology and Clinical Language Sciences at The University of Reading. All procedures involving human participants were approved by the University of Reading Ethics Committee. Written informed consent was obtained from all participants, and a tick box replaced the requirement for a signature in the online study.

### 2.2 Treatments

Participants received either a lactose placebo tablet, Vitamin B6 tablets, or Vitamin B12 tablets. The B12 tablets each contained 1000 μg of B12 as methylcobalmin, and the B6 tablets each contained 100 mg of B6 as pyroxidine hydrochloride. The tablets were donated by Innopure (https://www.innopure.com/). Because the different types of tablet were not visually identical they were transferred to medicine bottles that had been covered with black adhesive tape so that the tablets contained inside were not visible to the experimenters who handed them to the participants. For the 30–35 days of the study participants were asked to ingest one tablet per day with food. Bottles contained 40 tablets to allow for the possibility of a slightly longer supplementation period if this proved necessary; scheduling post‐tests lab visits was made more practical by this 5‐day flexibility window. Participants were asked to return the medicine bottles when they attended the lab for post‐tests and experimenters checked the tablets remaining in each bottle.

To double blind the study, after the medicine bottles were filled with tablets, each bottle was numbered, and the contents recorded. Numbers were allocated to bottles randomly using a computerised method by a PhD student from the PI's lab who was not part of the study. During the baseline session each participant selected a bottle from a mixed bag and the experimenter recorded the number on the bottle. When the study was adapted to continue online, instead of the participant selecting a bottle an experimenter selected at random a sealed envelope containing one of the bottles with the bottle number written on the outside and wrote the participant address on it.

### 2.3 Outcome measures

#### 2.3.1 Screen for adult anxiety related disorders (SCAARED)

The SCAARED is a factor‐analytically derived measure of anxiety symptoms in adults designed to be directly comparable to the Screen for Child Anxiety Related Emotional Disorders, for which high levels of reliability and validity have been established (Angulo et al., 2017). The SCAARED consists of 44 items and produces in a total score as well as four subscale scores: somatic/panic/agoraphobia (SCAARED‐SPA), generalised anxiety (SCAARED‐GAD), separation anxiety (SCAARED‐SEP), and social anxiety ,SA (SCAARED‐SA). Higher scores indicate more symptoms of anxiety.

#### 2.3.2 Mood and feelings questionnaire: Long version

The MFQ is a measure of depression symptoms with alternative versions for children, adolescents, and young adults (Costello & Angold, 1988). It has been extensively validated (e.g., Burleson Daviss et al., 2006). We used the adult self‐report version, which has 33 items that aggregate into a single score. Higher scores indicate more symptoms of depression.

#### 2.3.3 Visual contrast sensitivity and surround suppression

This task measures the minimum percentage contrast between the lighter and darker regions of a striped pattern (Gabor patch) that can be detected, called the contrast threshold, and was adapted from Petrov and McKee (2006). We also measured the contrast threshold in the presence of a suppressive surround mask; this increases the threshold and there is evidence that this effect is mediated by GABAergic connections in visual cortex (Kéri, 2015). Which version of the task was completed first was randomised between participants. Each trial began with the presentation of a central fixation cross on a grey background (brightness 25 cd/m2) and four black guide circles positioned above, below, and to the left and right of the fixation cross to indicate the four possible locations at which the target might appear. Each guide circle was centred on a visual eccentricity of 4.5° and had a diameter of 1.1°. The target (Gabor patch, oriented at 45°, spatial frequency 1.3 cycles per degree, mean luminance 25 cd/m2) was presented for 150 msec at one of the four possible locations, and to inform the participant it was being presented an auditory tone of the same duration was played simultaneously. The participant's task was to indicate which of the four target positions the target had appeared at by pressing one of the four arrow keys on the computer keyboard. On the first trial of the version of the task without the suppressive surround mask the target contrast was set to 14%, which is easily detected. Thereafter, the contrast of the target was adjusted using a 1‐up‐1‐down descending staircase procedure in which the target contrast was reduced if the participant responded correctly and increased if they made an error. The staircase continued until the participant had made three errors, at which point the contrast of the target that was presented in the final trial was taken as the estimate of the participant's contrast threshold. To make the descending staircase more efficient the initial reduction in target contrast for a correct answer was set to 2%, but the step size was reduced by 25% each time a correct response was made smaller as the contrast threshold was approached, subject to a minimum step size of 0.17%. The version of the task with the suppressive surround mask differed in only two respects. Firstly, the grey background was replaced by a striped pattern at 10% contrast, with the same orientation and spatial frequency as the target Gabor patches. This was displayed continuously throughout the task. The pattern did not extend inside the four guide circles. Secondly, because contrast thresholds are higher in the presence of a suppressive surround, the target contrast on the initial trial was set to 18%. In both versions of the task the participant's head was supported in a chin rest at a distance of 85 cm from a gamma corrected CRT monitor with a viewable window 32.5 cm wide and 24.5 cm high and a refresh rate of 100 Hz. A small number of practice trials were provided before the first task, which was performed in a darkened room. The experiment was programmed in Matlab, using Psychtoolbox (Brainard, 1997; Pelli, 1997). To achieve the low contrasts and small contrast increments necessary for the staircase procedure used here we utilised the ‘EnablePseudoGrayOutput’ option available in Psychtoolbox.

#### 2.3.4 Binocular rivalry

When different stimuli are presented to corresponding retinal locations of the two eyes, rather than perceiving a blend of the two stimuli, perception alternates between the two. It is thought that the two stimuli compete to suppress each other, and that this process relies on GABAergic inhibitory interactions between two populations of neurons in visual cortex (Mentch et al., 2019; Robertson et al., 2013). Following Van Loon et al. (2013), who used Magnetic Resonance Spectroscopy (MRS) to show that participants with higher GABA levels in visual cortex had slower alternation rates, we measured the perceptual alternation rate between two greyscale Gabor patches of orthogonal orientations. To allow us to present different stimuli to the two eyes the participant used alternating LCD shutter glasses and the same CRT monitor and chin rest as described above. This resulted in a refresh rate for each eye of 50 Hz. Both Gabor patches were presented in the centre of the screen and were defined by a diameter of 5°, a contrast of 50%, and a spatial frequency of 1 cycle per degree. One Gabor was tilted 45° leftwards away from vertical, and the other was tilted 45° rightwards away from vertical. To assist accurate convergence of the eyes at the correct distance, the Gabor patches were surrounded by a square made up of smaller squares whose contrasts with the background varied. The participant's task was to view the centre of the screen for 1 min and press the left arrow key if they clearly saw the Gabor patch tilting to the left, and the right arrow key for the opposite percept. During periods when they perceived a mixed or unclear percept then they were instructed to press neither key. There was an initial practice trial (1‐min) to familiarise with the task, followed by two 1‐min trials. The outcome measure was the average number of perceptual switches between the two orientations per minute.

#### 2.3.5 Tactile test battery

We used a ‘Brain Gauge pro’ device (https://www.corticalmetrics.com/), which consists of two 5 mm diameter cylindrical probes that stimulate the forefinger and middle finger of the participant's left hand to measure several aspects of vibrotactile sensory processing (Tommerdahl et al., 2019). These tests were included because previous research has suggested that some of them are related to inhibitory GABAergic processing, in particular in ASD (Puts et al., 2014; Puts et al., 2017; Tavassoli et al. (2015)). At the beginning of the vibrotactile test battery participants were asked to rest the forefinger and middle finger of their left hand on the left and right probes of the device, which then provided examples of suprathreshold vibrotactile stimulation of each finger. If at any point in the test battery the participant pressed too hard on the buttons they were reminded to reduce the pressure by an on‐screen prompt. The test battery consisted of the following measures, which were made in this order:

(1) *Simple reaction time*: this required the participant to press the left mouse button with their right hand as soon as vibrations were detected by their forefinger. The stimulus vibrated at 25 Hz, had an amplitude of 300 μm, and a duration of 40 msec. There was a practice trial followed by 10 trials separated by variable inter‐trial intervals. For each participant a truncated mean reaction time was calculated from 6 trials, excluding the two fastest reactions and the two slowest. Intrasubject variability in reaction time was also calculated as the SD across all ten trials.
(2) *Static threshold*: this test measured the minimum amplitude of vibration that was detectable, and used a two alternative forced choice paradigm with a descending staircase. The stimulus on the first trial (20 μm, 25 Hz, 500 msec) was delivered to either the forefinger or the middle finger and the participant had to respond by using the mouse to indicate ‘left’ or ‘right’. For the first 10 trials the amplitude (µm) was adjusted using a 1‐up‐1‐down staircase, and for the final 10 trials a 2‐up‐1‐down staircase was used. The threshold was calculated as the mean amplitude of the final five trials. Participants were instructed to guess if they were unsure, and were required to get three practice trials in a row correct before moving on to the block of twenty trials that measured the threshold.
(3) *Dynamic threshold*: this differed from the ‘static threshold’ in that the stimulation amplitude began at an undetectable level and gradually increased until the participant detected it. On each trial a continuous 25 Hz vibration began with an amplitude of 0 μm and increased at a rate of 2 μm/s until the participant was able to detect it, at which point they used the mouse to indicate whether the stimulus had been delivered to the forefinger or the middle finger. There were 7 trials and the threshold was calculated as the mean of the stimulation amplitudes at the times of pressing the mouse button, excluding incorrect trials. We asked participants to refrain from responding until they were confident of correctly identifying which finger was being stimulated. There was one practice trial. ‘Dynamic thresholds’ are typically higher than ‘static thresholds’, which is thought to occur because of feedforward inhibition (Robertson & Baron‐Cohen, 2017); interestingly, in ASD the difference between the two threshold types is reduced or absent (Puts et al., 2014; Tavassoli et al., 2015). Therefore, we also calculated and analysed the percentage increase in the threshold measured dynamically (dynamic‐static/static).
(4) *Single Site Adapted Discrimination Threshold (SSADT)*: this test measured the just noticeable difference (JND, also known as the discrimination threshold) in amplitude between an easily detectable Standard (200 μm, 25 Hz, 500 msec) and a variable Comparison (initial values 400 μm, 25 Hz, 500 msec). The two stimuli were presented simultaneously, one to each finger, and the participant used the mouse to indicate which of the two they perceived to be weaker. The Comparison was preceded by an adapting stimulus (200 μm, 25 Hz, 1000 msec) that acts to reduce its perceived amplitude (Tannan et al., 2007). For the first 10 trials the amplitude (µm) of the Comparison was adjusted using a 1‐up‐1‐down staircase, and for the final 11 trials a 2‐up‐1‐down staircase was used. Participants were instructed to guess if they were unsure, and were required to get three practice trials in a row correct in order to move on to the block of twenty‐one trials that measured the threshold. The SSADT was calculated as the mean amplitude of the Comparison stimulus on the final five trials. This score represented how much stronger the Comparison stimulus had to be than the Standard for the difference between the two to reliably be detected, and the effect of the adapting stimulus was to increase the required difference.

#### 2.3.6 Procedure

Data were collected in five separate phases over a period of approximately 4.5 years. The treatment regime was identical in all phases, but details of the testing procedure varied. Table 2 describes the order of tests and other procedures at baseline and post‐test in each of the five phases, including those tests for which methodological details and results will be reported in a subsequent manuscript when a sixth and final phase of data collection has been completed. Testing for phases 1–4 was carried out in the lab of the corresponding author, but due to the COVID‐19 pandemic phase 5 took place online (bottles of vitamin tablets were posted to participants). The online version of the experiment was constructed using the Gorilla Experiment Builder (www.gorilla.sc; Anwyl‐Irvine, Massonié, Flitton, Kirkham, Evershed, 2019) and the ongoing phase 6 also uses the online testing methodology. For practical reasons it was not always possible to ensure that post‐tests took place at the same time of day as the baseline measurements, although the difference in starting times was minimised where possible and was often small. In phases 1‐4 testing always took place between 08.30 and 18.00 on week days, but for the online study the exact time of testing was under the control of the participant.

**TABLE 2:** Overview of the procedures in the 5 phases of the study

Timeline	Phase 1	Phase 2	Phase 3	Phase 4	Phase 5
Recruitment	Study adverts placed on local SONA student research panel, social media, and physical notice boards. Initial eligibility of potential participants assessed by email.				Researchers met respondents to the study advert in a video call to screen them; tablet bottle posted; participant contacts researcher when bottle received; researcher sends link to baseline test battery
	153 participants	88 participants	81 participants	103 participants	53 participants
Baseline procedure, varying in duration between 5 min in phase 1 and 50 min in phases 4 and 5	Screening questionnaire	DASS	DASS	SCAARED	Current state covariates
		MFQ	MFQ	DASS	Dietary info.
		SCAARED	SCAARED	AQ10	HRV
		ASRS	PSQI	SP3D	Ebbinghaus illusion magnitude
		AQ10	SP3D	ACS	Tilt illusion magnitude
		PSQI	ASRS	ASRS	GoNoGo
		SP3D	AQ10	Dietary info.	AQ10
				Binocular rivalry	SP3D
				CS suppression	SCAARED
				Tactile battery	ACS
				GoNoGo	ASRS
				HRV	DASS
End of first lab visit	Participant selects tablet bottle at random				Not applicable
Approx. 15 days after 1^st^ lab visit	Email reminding participants about tablets and to attend post‐test session				
30 days after baseline	Not applicable				Gorilla website sends weblink (URL) to participants for post‐test measures
Post‐test procedure, 30–35 days after baseline	Bottle returned	Bottle returned	Bottle returned	Bottle returned	Current state covariates
	CS suppression	Binocular rivalry	Binocular rivalry	SCAARED	HRV
	Dietary info.	CS suppression	CS suppression	DASS	Ebbinghaus illusion magnitude
	AQ10	Tactile battery	Tactile battery	AQ10	Tilt illusion magnitude
		DASS	DASS	SP3D	GoNoGo
		MFQ	MFQ	ACS	AQ10
		SCAARED	SCAARED	ASRS	SP3D
		PSQI	PSQI	Dietary info.	SCAARED
		ACS	ASRS	Binocular rivalry	ACS
		Dietary info.	Dietary info.	CS suppression	ASRS
				Tactile battery	DASS
				GoNoGo	
				HRV	

*Note*: Time elapsed runs from top to bottom. Tests were conducted in the order listed. Results from the tests in bold font are reported in this article. Results from the other tests will be reported in a follow‐up article.

Abbreviations: ACS, Attentional Control Scale; AQ10, Autism Spectrum Quotient; ASRS, Adult ASRS Self‐Report Scale version 1.1; CS suppression, Visual Contrast Sensitivity with and without spatial suppression; DASS, Depression, Anxiety, and Stress scale (42 item version); HRV, Heart Rate Variability; MFQ, Mood and Feelings Questionnaire; PSQI, Pittsburgh Sleep Quality Index; SCAARED, Screen for Adult Anxiety Related Disorders; SP3D, Sensory Processing 3 dimensions scale.

#### 2.3.7 Data processing and statistics

We excluded participants from statistical analyses of the mental health questionnaires if scores on outcome variables were extreme or implausible, implying that the questionnaire instructions were not followed correctly. We defined ‘implausible’ as scoring higher than the highest scores obtained in a clinically diagnosed sample. This was done separately for the SCAARED and the MFQ. In the case of the SCAARED anxiety questionnaire, we excluded participants with total scores > 61 at either baseline or post‐test on the grounds that in an investigation of the psychometric properties of the SCAARED that included a patient group with 55 anxiety patients, the highest scoring patient scored 61 (Angulo et al., 2017). This resulted in the exclusion of 12 participants at baseline and 12 participants at post‐test. In the case of the MFQ depression questionnaire, we excluded participants with total scores > 37 at either baseline or post‐test on the grounds that the mean MFQ score in a sample of 77 patients undergoing major depressive episodes was found to be 32.8 (Burleson Daviss et al., 2006); participants with scores genuinely this high would be unable to take part in the study. This resulted in the exclusion of 3 participants at baseline and 6 participants at post‐test. These exclusions are reflected in the sample sizes presented in Table 1.

We excluded participants from statistical analysis of the Visual Contrast Sensitivity task if they failed to show the basic surround suppression effect, that is, their threshold was lower in the presence of the suppressive surround. Such a pattern of performance is likely to indicate inattention or that the task instructions were misunderstood. Following these exclusions we defined outliers in the contrast thresholds measured with and without the suppressive surround using the outlier labelling rule (Hoaglin & Iglewicz, 1987), which defines outliers as those scores lying 2.2 * the interquartile range or further from the 1^st^ or 3^rd^ quartile. This second step acted to exclude participants whose measured contrast thresholds were unrealistically high, suggesting inattentiveness. Finally, we checked for participants whose thresholds with and without the suppressive surround were not extreme, but who showed an excessively large difference between the two, which would indicate inattention during one of the measurements; we found one such participant, whose threshold with surround was more than three times greater than without. These exclusions are reflected in the sample sizes presented in Table 1.

We excluded a small number of participants from statistical analysis of the Binocular Rivalry task who reported only zero or one perceptual reversals per minute. Such a pattern of performance is likely to indicate inattention or that the task instructions were misunderstood, or alternatively could indicate extreme eye dominance (e.g. amblyopia). Following these exclusions we defined outliers using the outlier labelling rule (Hoaglin & Iglewicz, 1987). This second step acted to exclude one participant with a very high reversal rate. These exclusions are reflected in the sample sizes presented in Table 1.

We excluded participants on a pairwise basis from the statistical analysis of the various measures making up the Tactile Test Battery using the outlier labelling rule (Hoaglin & Iglewicz, 1987). Additionally, for the dynamic threshold measurement, because participants were instructed not to respond until the intensity of vibrotactile stimulation had risen to the point where they were certain which finger was being stimulated, a low percentage correct indicated failure to follow the task instructions. Therefore, prior to application of the outlier labelling rule participants were excluded if performance was less than 70% correct. This second step also reduced the sample size for the analysis of the difference between static and dynamic thresholds. These exclusions are reflected in the sample sizes presented in Table 1.

We analysed the total scores and subscales of the mental health questionnaires using a pair of 2 (baseline vs. post‐test) by 2 (treatment group) mixed ANOVAs. One ANOVA was run to compare B6 with placebo and one was run to compare B12 with placebo. We followed these up with paired samples t‐tests assessing change between baseline and post‐test within each treatment group. We analysed the contrast sensitivity and surround suppression scores using a pair of 2 (surround absent vs. surround present) by 2 (placebo vs. either B6 or B12) ANOVAs, and followed these up with independent samples t‐tests comparing treatment groups within surround condition. The binocular rivalry and tactile test battery scores were analysed using independent samples t‐tests comparing the Vitamin B6 group scores to placebo, and comparing the Vitamin B12 group scores to placebo. All *p* values reported are two‐tailed.

## 3 RESULTS

Prior to analysis of the data potential differences in age and gender between treatment groups were assessed by ANOVA. Because the sample differed between outcome variables, this analysis was repeated for each outcome variable. No significant differences were found.

### 3.1 Screen for adult anxiety related disorders (SCAARED)

**FIGURE 1:**
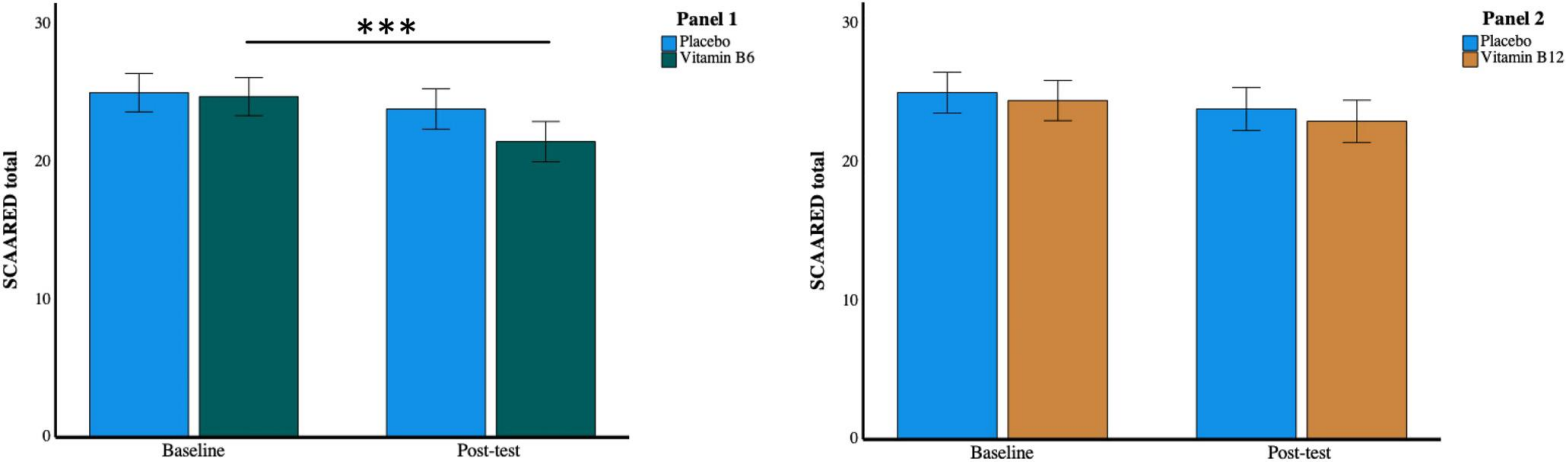
Panel 1: the reduction in anxiety (SCAARED questionnaire total scores) in the Vitamin B6 group between baseline and post‐test; Panel 2: the trend towards a reduction in anxiety in the Vitamin B12 group. Symbols above bars indicate significant differences between baseline and post‐test, *p* < 0.001. Error bars indicate +/− 1 SEM

Figure 1 shows the baseline and post‐test mean (±SE) SCAARED total scores for each vitamin group compared to the placebo group. The ANOVA analysing the B6 and placebo group data revealed a highly significant reduction in anxiety at post‐test (F(1,173) = 10.03, *p* = 0.002, *η*_*p*_^2^ = 0.055). This was driven mainly by reduced anxiety in the B6 group (t(88) = 3.51, *p* < 0.001, *d* = 0.37), while the smaller reduction that occurred in the placebo group was non‐significant (t (86) = 1.21, *p* = 0.265, *d* = 0.12). Despite this dissociation, the interaction did not reach significance (F(1,173) = 2.20, *p* = 0.140, *η*_*p*_^2^ = 0.013). The ANOVA comparing the B12 group to placebo revealed a significant reduction in anxiety at post‐test (F (1,175) = 4.08, *p* = 0.045, *η*_*p*_^2^ = 0.023). This was driven mainly by a trend towards reduced anxiety in the B12 group (t(89) = 1.84, *p* = 0.069, *d* = 0.19), but the interaction was not significant (F (1,17) = 0.06, *p* = 0.812, *η*_*p*_^2^ < 0.001).

We also examined whether the effects of treatment group differed between the subscales of the SCAARED using t‐tests comparing baseline and post‐test scores. Table 3 presents the results of this, along with descriptive statistics. The B6 group reported a highly significant reduction on the GAD (generalised anxiety disorder) subscale, as well as a significant reduction on the SA subscale, and trends towards reductions on the other subscales. The B12 group reported a significant reduction on the SEP subscale (separation anxiety). In the placebo group, there were no significant changes.

**TABLE 3:** The effect of treatment group on change in anxiety scores between baseline and post‐test, broken down by anxiety subtype

Treatment	SCAARED subscale	Baseline mean (SD)	Post‐test mean (SD)	t	df	*p*	*d*
B6	Total	24.8 (12.8)	21.5 (12.7)	3.51	87	<0.001**	0.37
	SPA	6.1 (4.9)	5.4 (4.5)	1.96	87	0.053	0.21
	GAD	11.6 (5.9)	10.1 (6.0)	3.32	87	0.001**	0.35
	SEP	2.0 (2.4)	1.7 (2.1)	1.74	87	0.085	0.19
	SA	4.9 (3.8)	4.3 (3.8)	2.60	87	0.011*	0.28
B12	Total	24.5 (14.3)	23.0 (14.4)	1.84	89	0.069	0.19
	SPA	6.1 (4.9)	5.73 (5.5)	1.20	89	0.233	0.13
	GAD	10.9 (6.3)	10.47 (6.4)	1.15	89	0.255	0.12
	SEP	2.4 (2.5)	2.03 (2.4)	1.98	89	0.05*	0.21
	SA	4.9 (3.9)	4.74 (3.3)	0.53	89	0.597	0.06
Placebo	Total	25.1 (13.2)	23.9 (14.6)	1.21	86	0.265	0.12
	SPA	6.2 (5.2)	5.7 (5.3)	1.29	86	0.200	0.14
	GAD	11.6 (6.5)	11.1 (6.7)	0.90	86	0.370	0.10
	SEP	2.3 (2.3)	2.6 (2.7)	−1.51	86	0.135	−0.16
	SA	4.9 (3.2)	4.5 (3.7)	1.32	86	0.189	0.14

Abbreviations: GAD, generalised anxiety; SA, social anxiety; SEP, separation anxiety; SPA, somatic/panic/agoraphobia.

### 3.2 Mood and feelings questionnaire: Long version

**FIGURE 2:**
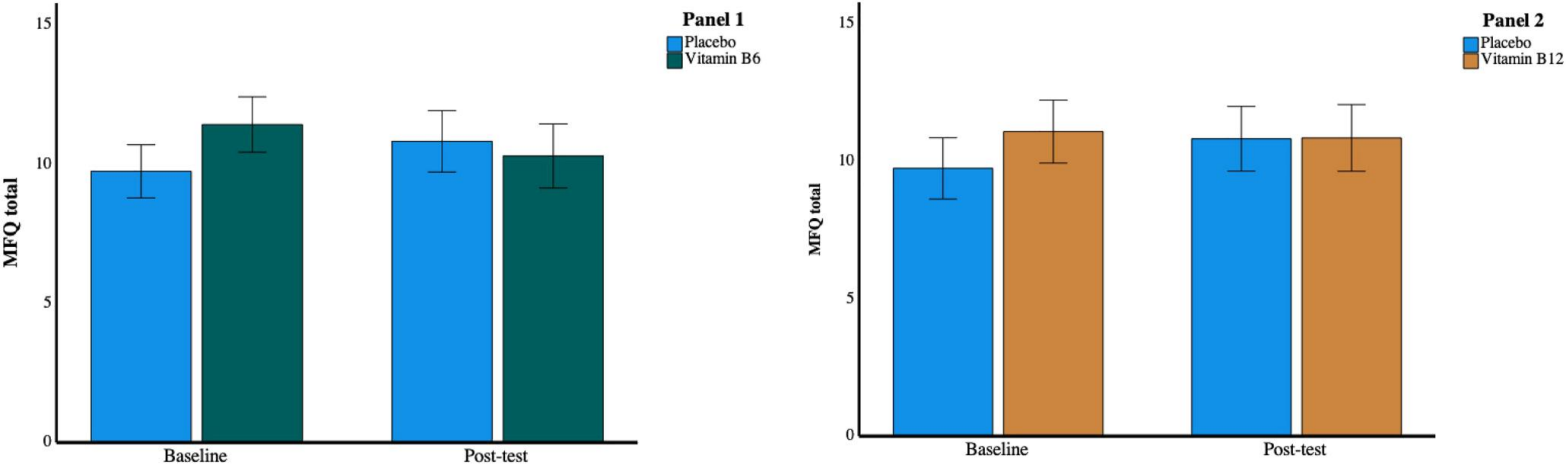
Panel 1: Comparing B6 to placebo, there was a trend towards an interaction between treatment group and direction of baseline to post‐test change in depression scores, which was not observed when comparing B12 to placebo (Panel 2) Error bars indicate +/− 1 SEM

Figure 2 presents the baseline and post‐test mean (±SE) MFQ total scores broken down by treatment group. The ANOVA analysing the B6 and placebo group data revealed no uniform direction of change in depression at post‐test (F(1,96) = 0.002, *p* = 0.969, *η*_*p*_^2^ < 0.001). Instead, there was a tendency for depression scores to decrease between baseline and post‐test in the B6 group but to increase in the placebo group, and this interaction approached significance (F(1,96) = 3.08, *p* = 0.083, *η*_*p*_^2^ = 0.031). However, follow up t‐tests comparing the baseline and post‐test scores within each of the treatment groups revealed no significant changes: B6 (t(46) = 1.13, *p* = 0.263, *d* = 0.17); placebo (t(50) = −1.37, *p* = 0.176, *d* = −0.19). The ANOVA analysing the B12 and placebo group data revealed no significant or trending effects, and the t‐test comparing the baseline and post‐test scores in the B12 group was non‐significant B12 (t(46) = 0.20, *p* = 0.843, *d* = 0.03).

### 3.3 Visual contrast sensitivity and surround suppression

**FIGURE 3:**
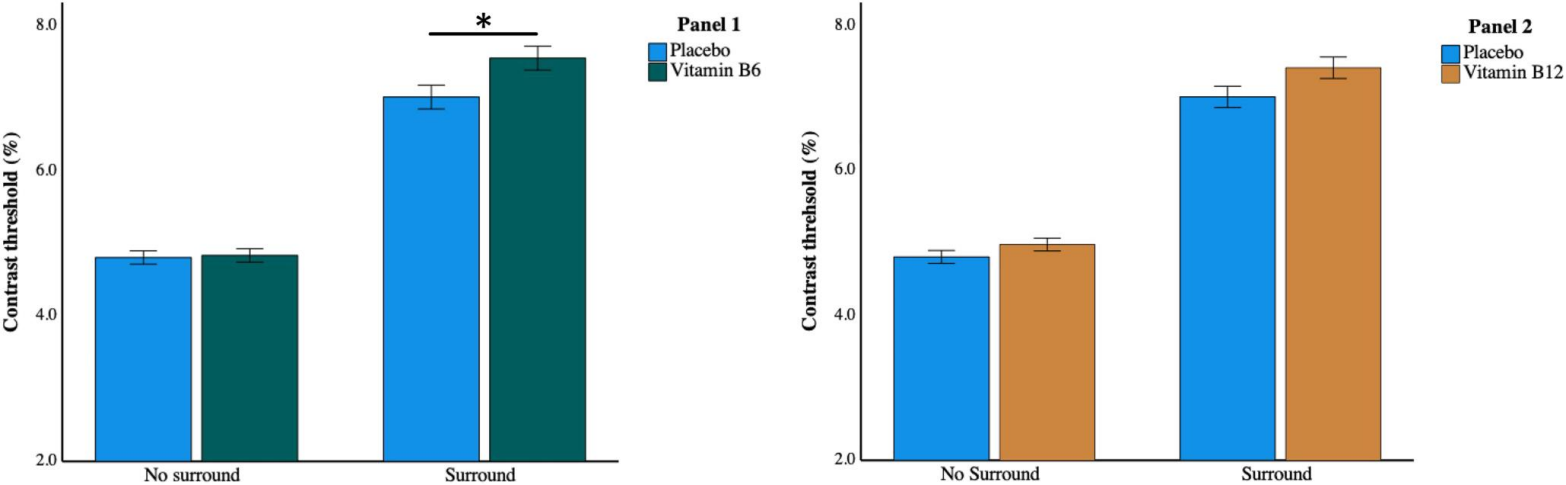
Panel 1: Vitamin B6 supplementation raised contrast thresholds significantly only in the presence of a suppressive surround; Panel 2: Vitamin B12 supplementation resulted a trend towards raised contrast thresholds. Symbols above bars indicate significant differences between baseline and post‐test, *p* < 0.05. Error bars indicate +/− 1 SEM

Figure 3 shows contrast thresholds without with a suppressive surround masks, comparing the performance of participants supplemented with B6 or B12 for a month prior to the measurements with that of the placebo group. There was a stronger effect of the suppressive surround in the B6 group, reflected in the significant interaction shown in Panel 1 between treatment group and the presence/absence of the surround (F(1,202) = 6.58, *p* = 0.011, *η*_*p*_^2^ = 0.031). Follow up t‐tests confirmed that there was a significant difference in the minimum amount of contrast that could be detected between the two groups when the surround was present (t(202) = −2.31, *p* = 0.022, *d* = −0.32), but not when it was absent (t(202) = −0.22, *p* = 0.828, *d* = −0.03).

Comparing the performance of participants supplemented with B12 for a month prior to the measurements with that of the placebo group, thresholds were generally elevated in the B12 group, whether or not a suppressive surround was present. This was reflected in a trend towards the significant main effect of treatment group shown in Panel 2 (F(1,204) = 3.72, *p* = 0.055, *η*_*p*_^2^ = 0.018). Although this effect was slightly larger in the presence of a suppressive surround, the interaction did not reach significance (F(1,204) = 1.68, *p* = 0.197, *η*_*p*_^2^ = 0.008). Follow up t‐tests showed a trend towards a difference in the minimum amount of contrast that could be detected between the two groups when the surround was present (t(204) = −2.31, *p* = 0.057, *d* = −0.27), which was non‐significant when it was absent (t(204) = −1.35 *p* = 0.179, *d* = −0.19).

### 3.4 Binocular rivalry

**FIGURE 4:**
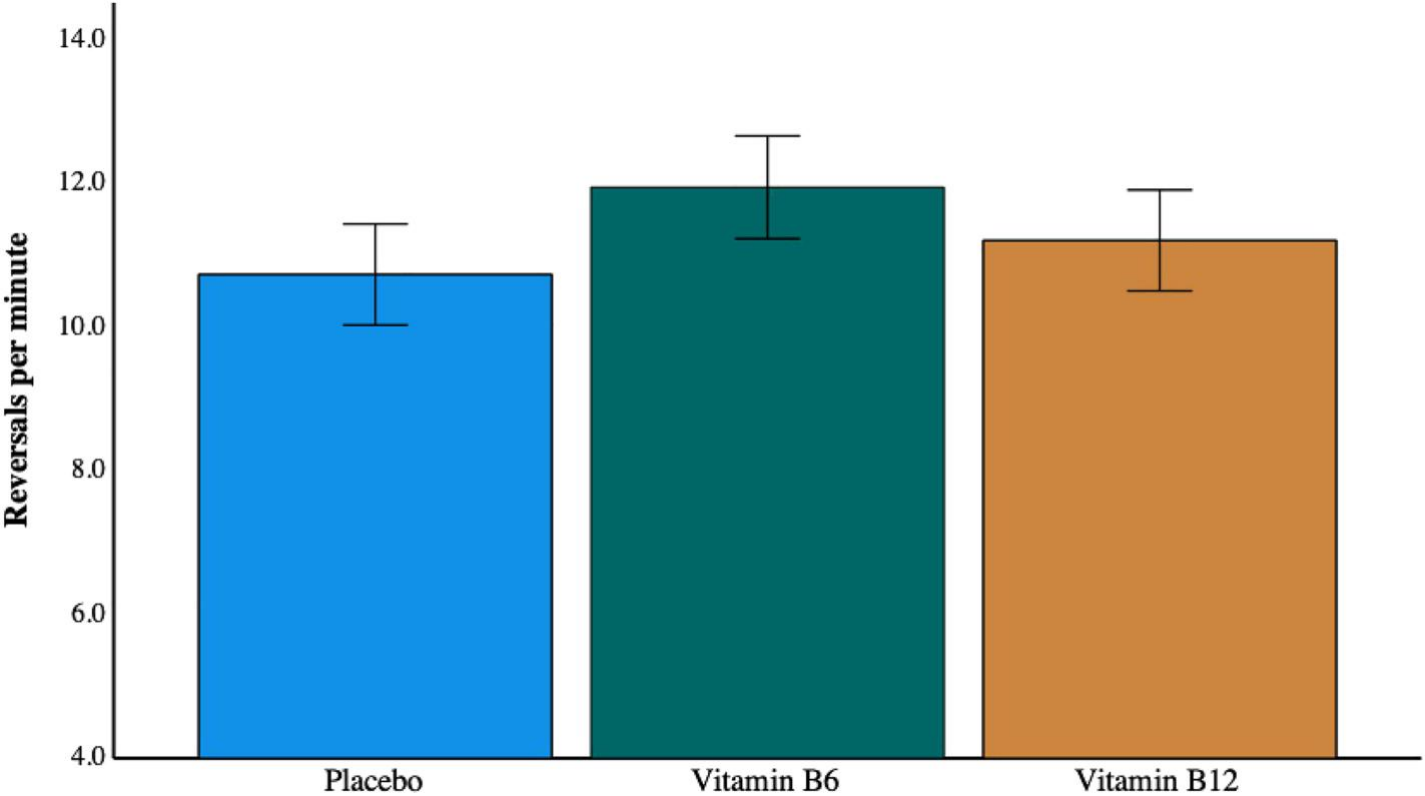
The non‐significant effect of treatment group on binocular rivalry reversal rates. Error bars indicate +/− 1 SEM

Figure 4 presents the number of perceptual reversals per minute, comparing the performance of the three treatment groups. Neither vitamin group differed significantly from placebo: B6 (t(112) = 1.2 *p* = 0.232, *d* = 0.23), B12 (t(114) = 0.466 *p* = 0.64, *d* = 0.09).

### 3.5 Tactile test battery

Neither vitamin group differed significantly from placebo on any of the six measures arising from the tactile test battery. However, we observed a trend towards lower scores on the SSADT in the B6 group, which if confirmed would indicate increased sensitivity to subtle differences between the intensities of tactile stimuli. There was also a trend towards greater intrasubject variability in reaction times in the B12 group relative to placebo. Details of these results are presented in Table 4.

**TABLE 4:** The effect of treatment group on components of the tactile sensory processing test battery

Treatment	Measure	Placebo mean (SD)	Vitamin mean (SD)	t	df	*p*	*d*
B6 versus placebo	Reaction time (msec)	277 (71)	270 (60)	0.63	151	0.528	0.10
	Rtm. variability (msec)	28 (18)	28 (17)	−0.01	150	0.989	−0.00
	Threshold, static (µm)	11.5 (4.3)	12.5 (4.8)	−1.45	155	0.149	−0.23
	Threshold, dynamic (µm)	14.8 (6.6)	15.9 (6.1)	−1.00	121	0.319	−0.18
	ThreshIncrease (%)	34.5 (56.7)	41.8 (44.3)	−0.79	120	0.433	−0.14
	SSADT (µm)	131.5 (68.6)	112.8 (60.8)	1.76	147	0.081	0.29
B12 versus placebo	Reaction time (msec)	277 (71)	274 (61)	0.34	151	0.732	0.06
	Rtm. variability (msec)	28 (18)	33 (22)	−1.71	152	0.089	−0.28
	Threshold, static (µm)	11.5 (4.3)	11.89 (3.7)	−0.63	155	0.531	−0.10
	Threshold, dynamic (µm)	14.8 (6.6)	15.5 (6.1)	−0.63	123	0.530	−0.11
	ThreshIncrease (%)	34.5 (56.7)	44.3 (60.7)	−0.93	122	0.335	−0.17
	SSADT (µm)	131.5 (68.6)	123.3 (76.3)	0.69	151	0.489	0.11

*Note*: ThreshIncrease (Dynamic threshold‐Static threshold)/Static threshold.

Abbreviation: SSADT, Single site adapted discrimination threshold.

## 4 DISCUSSION

We evaluated whether the balance between neural excitation and inhibition, as assessed by a range of behavioural outcome measures, could be altered through supplementation with either Vitamin B6 or B12 relative to placebo. In a general population sample skewed towards younger, female, adults our two key findings both suggest that supplementation with Vitamin B6 influences the balance between excitation and inhibition. Firstly, B6 supplementation increased visual contrast thresholds when a suppressive surround was present, but this did not occur in the absence of the surround. This finding points very specifically to an increase in neural inhibition by increasing GABA levels because the effect of the suppressive surround on thresholds is caused at least in part by the action of inhibitory GABAergic interneurons (Spiegel et al., 2012; Yoon et al., 2010). On the other hand, if the other potential actions of B6 in the brain had been responsible this would also have elevated thresholds when the surround was absent, because those mechanisms act to reduce excitation. Consistent with this interpretation, the pattern of results produced by Vitamin B6 supplementation in this visual paradigm is the same as that produced by the GABA agonist alprazolam (Kéri, 2015; Yoon et al., 2010). Also supporting the interpretation that B6 influenced performance by altering GABA levels is the finding that GABA levels in visual cortex measured by MRS predict performance when a surround is present but not when it is absent (Yoon et al., 2010). Our second key finding was that B6 supplementation reduced self‐reported anxiety symptoms, with a larger effect size for GAD symptoms. Dysfunction of the GABA system has long been associated with anxiety; drugs that positively modulate GABA receptors are generally anxiolytic, and drugs that negatively modulate GABA receptors are generally anxiogenic (Kalueff & Nutt, 2007; Nemeroff, 2003). This suggests that an increase in GABA levels explains the reduction we found in anxiety, or this mechanism may offer a partial explanation with additional contributions being made by the three potential pathways to reduced neural excitation described in the Introduction. In contrast, we found no clear effects of B6 supplementation on other outcome measures previously linked to the balance between excitation and inhibition; these were self‐reported depression symptoms, binocular rivalry reversal rate, and the tactile test battery. However, we note that the statistical power of the study to detect small effect sizes was more limited for these outcome measures.

We argued that Vitamin B12 supplementation might indirectly influence the balance between excitation and inhibition via its role in myelination because a high proportion of local inhibitory neurons are myelinated (Micheva et al., 2016), or by increasing the rate at which homocysteine is recycled to cysteine, but the results of the study neither support this proposal nor rule it out. We found no clearly significant effects of Vitamin B12 supplementation on the outcome measures, although there was a suggestive trend towards reduced anxiety, and a trend towards an increase in visual contrast thresholds. B12 supplementation also produced an unexpected trend towards greater intrasubject reaction time variability. These equivocal results stand in contrast to the results of a recent study of B12 supplementation in well‐nourished young adult rats, which found a clear effect in the form of a large increase in the somatosensory evoked local field potential (Kang et al., 2019). It is possible that the 1 month supplementation period in the present study was insufficient for the effects of B12 supplementation, particularly those relating to myelination, to fully emerge. While a study in mice found effects of B12 on re‐myelination following traumatic brain injury in mice within two weeks (Wu et al., 2019), and Kang et al. (2019) found an effect in rats within 1 month, the limited human literature looking at the effects of B12 supplementation in clinical case studies in which demyelination is present suggests that several months of treatment is required to achieve results(e.g., Senol et al., 2008; Timms et al., 1993).

As well as suggesting that Vitamin B6 supplementation acted to increase GABAergic inhibition, the finding that B6 elevates contrast thresholds in the presence of a suppressive surround is significant in several other ways. Firstly, like the Marmite supplementation study of Smith et al. (2017) our results indicate a reduction in excitatory responses and so our findings constitute a replication of that study, but with some caveats; while our results can be attributed directly to Vitamin B6, marmite contains relatively little B6 but does contain high levels of B12 and other B vitamins, as well as other ingredients such as tryptophan (a serotonin precursor), suggesting that an increase in neural inhibition can be achieved through multiple dietary pathways. Secondly, the fact that contrast thresholds were elevated, which corresponds to worse task performance, rules out domain‐general mechanistic explanations of the effects of the vitamin supplement such as the idea that B6 supplementation made participants feel more alert or attentive. Thirdly, it would be incorrect to conclude from the elevation of contrast thresholds in the presence of a suppressive background that B6 supplementation would be detrimental to vision in an everyday context. This is because our laboratory task measured visual performance in the unusual situation in which the target to be detected and the background are identical in most respects. The lateral inhibitory connections in the visual cortex that produce the elevated thresholds in this paradigm are spatial frequency and orientation tuned (Polat & Sagi, 1993), and consequently if the visual background differs from what the Observer attends to then suppression does not occur. In fact, under most viewing conditions lateral inhibitory processes act to sharpen the neural contrast between salient features of the retinal image such as edges and the background (Yantis & Abrams, 2016).

The finding that Vitamin B6 supplementation reduced self‐reported anxiety is consistent with the findings of a recent large cross‐sectional study which found that higher B6 intake is associated with lower anxiety risk in women, though not in men (Kafeshani et al., 2020). In contrast, the two previous supplementation studies we are aware of that included a B6 supplementation group did not find improvements in mood related measures, but did find improvements in memory in older males and older (but not younger) women (Bryan et al., 2002; Deijen et al., 1992). Potential reasons for this disparity include differences between the populations studied, that the mood measures used made a less granular assessment of anxiety symptoms than the SCAARED questionnaire we used, and that our larger sample size provided more statistical power than the previous studies. Furthermore, it was noteworthy that the average level of self‐reported anxiety at baseline in our sample was high, including many participants who were potentially experiencing an anxiety disorder; it is possible that the benefits of supplementation for anxiety were more evident in our study because of this. Our continuing data collection may allow us to determine whether the benefits of supplementation depend on baseline anxiety status. In the Introduction we noted that a number of studies have found positive effects on measures of stress and anxiety following supplementation with multivitamin tablets containing B‐vitamins. Our findings suggest that Vitamin B6 was one of the main drivers of those findings, an idea that is consistent with the finding that benefits of multivitamin supplementation for mood were modulated by the B6 status of participants (Long & Benton, 2013). Our working hypothesis is that the role of B6 in GABA metabolism offers a complete or partial explanation of the effects we found; future studies could test this more directly using Magnetic Resonance Spectroscopy (MRS).

In contrast to anxiety, we found only a trend towards an effect of Vitamin B6 on self‐reported depression symptoms. The statistical power of the study to detect an effect of B6 on depression was lower than for anxiety, which may account for this. From a theoretical perspective an effect was expected because the two conditions are intrinsically related and overlap in terms of GABAergic neurochemistry; interventions that are anxiolytic also reduce depression symptoms (Kalueff & Nutt, 2007). Given this, another potential reason that we found an effect of B6 on anxiety but only a trend for depression is that in the present study, relative to clinical diagnostic cut‐offs, average baseline levels of anxiety were considerably higher than those of depression. To resolve this, future studies should determine if Vitamin B6 supplementation is more effective at reducing symptoms when these are initially greater.

We found no effect of supplementation with either vitamin on the Binocular Rivalry perceptual reversal rate, suggesting that there is no large effect of supplementation on this outcome variable, though we can't rule out small effects that might be detected in a larger study. The methodology should be improved in future studies by introducing a direct measurement of the time for which the percept was mixed or unclear. This can be achieved by introducing a 3^rd^ response button which allows calculation of a ‘suppression index’, where more suppression corresponds to a greater proportion of time for which a clear percept is perceived. This methodology was used by Robertson et al. (2016) to demonstrate using MRS that higher GABA levels in visual cortex predict stronger perceptual suppression, as well as by Mentch et al. (2019) to show that perceptual suppression is increased by drugs that modulate GABA receptors: clobazam (GABAA) and arbaclofen (GABAB).

We found no clear effect of supplementation with either vitamin on any component of the tactile test battery. Two caveats to this results are the lack of power to detect small effect sizes and poor compliance with task instructions by many participants when the threshold was measured dynamically; correctly following the instructions by waiting until the stimulus ramps up to the point where it is clearly detectable results in 100% correct performance, but many of our participants achieved lower performance levels than this, which is problematic because it implies that their response times on ‘correct’ trials might be artificially low due to contamination by ‘correct guesses’.

This exploratory study had a number of methodological limitations, and the key findings would be strengthened by a replication that addressed the following issues. We were not able to use blood serum analysis to assess baseline and post‐test levels of B6 and B12. Such measurements would verify participant compliance and that the supplements were effective in raising serum vitamin levels. The lack of such measurements in the current study is more relevant to the interpretation of the lack of significant effects of B12, which leaves open the question of how successful we were in raising B12 serum levels. On the other hand, because we found reliable effects of B6 we can be confident that B6 supplementation was, on average, successful in raising serum levels. Nonetheless, it is likely that our sample contained some participants we were unable to exclude whose compliance with the supplementation regime was poor; the influence of this would be to reduce the effect sizes we found. Secondly, the performance of a significant minority (almost 10%) of participants in the psychophysical tasks (e.g. contrast sensitivity) was surprisingly poor or unusual, and these participants were excluded on the assumption that this reflected inattention to the tasks or failure to understand instructions. However, if any of the unusual patterns of performance we observed‐such as contrast thresholds being superior with a surround than without‐were genuine and replicable in those individuals, then this would indicate the existence of subpopulations that we can't generalise our current finding to. Thirdly, because the experiment was wide ranging and exploratory, and included a large number of outcome variables, adjustments for multiple statistical comparisons were not made in order to minimise Type II error (false negatives). We performed 42 inferential tests, of which 6 rejected the null hypothesis at alpha 0.05. If the null hypothesis was true, and assuming the tests were independent, then on average across many replications the null hypothesis would be rejected only 2.1 times per experiment. This suggests both that the six rejections of the null we report reflect real underlying effects, and that we may be reporting one or more false positives. We note that this analysis is probably conservative because the 42 tests we performed were of correlated rather than independent outcome variables, and were underpinned by fewer than 42 independent null hypotheses. Finally, the sample sizes for the MFQ, binocular rivalry, and tactile tests, were not sufficiently large to detect small effect sizes and so type II errors can't be ruled out.

In conclusion, we have shown that supplementation with a high dose of a single vitamin (B6) can influence behavioural outcomes such as self‐reported anxiety. This approach is more effective than multivitamin studies for identifying candidate mechanisms. In the case of B6, we also found that it increased the surround suppression of visual contrast detection, which argues for an inhibitory GABA related underlying mechanism. Given that surround suppression is found to be abnormal in many patient groups, this suggests that the efficacy of high‐dose Vitamin B6 should be determined in these groups; for example, Vitamin B2 (riboflavin) is effective as a treatment for severe migraine (Chen et al., 2021), but also one RCT found that B6 given alone is also effective for migraine with visual symptoms, and several other RCT's have produced positive results for B6 as part of a combination supplement for migraine (Liampas et al., 2020). Furthermore, measuring the effects of other candidate micronutrients on the same outcome variables as those employed here may identify a list of micronutrients that could be combined and tested as a ‘nutritional psychiatry’ treatment for conditions such as anxiety and depression.

## CONFLICT OF INTEREST

No conflicts of interest have been declared.

## Data Availability

The data that support the findings of this study are available from the corresponding author upon reasonable request.
